# Clinical and pathological correlations in endometrial pathology

**Published:** 2015

**Authors:** RE Bohîlțea, M Sajin, F Furtunescu, LC Bohîlțea, A Mihart, A Baros, AF Anca

**Affiliations:** * “Carol Davila” University of Medicine and Pharmacy, Bucharest, Romania

**Keywords:** clinical and pathological correlations, endometrial cancer, abnormal uterine bleeding, endometrial carcinoma

## Abstract

The incidence and mortality rate of endometrial cancer has been registering an increasing trend both in Romania and in the whole world. The paper’s aim is to analyze the diagnostic approach of endometrial pathology in the University Emergency Hospital Bucharest, on a four years period. The medium age of the patients was of 50.51 ± 10.924 years, and the median age was of 48 years. The youngest patient suffering from endometrial cancer was of 30 years. Dilation and uterine curettage represent the main method used in the performance of endometrial biopsy, based on which the certitude etiologic histopathologic diagnosis was established in 68.4% of the patients with endometrial pathology. Hyperplasias represented half of the pathology (54.9%), most of them being without atypias. Endometrial carcinoma was identified in 19% of the patients. The diagnosis of the disease in IA stage represents 5.5% of the total endometrial cases and the diagnosis of the disease in the stage of its limitation to the uterus (stage IA, IB and IC) was of 64.2%. The endometrioid adenocarcinoma represents the most encountered histopathological form and the degree of tumor differentiation established for 68,15% of the cases was predominantly 1 and 2 (88%). The main symptom, which determines the patients’ decision to go to the physician, is the abnormal uterine bleeding. 66% of the cases of endometrial cancer in the stage of the disease limited to the uterus are diagnosed in Romania based on the abnormal uterine bleeding. However, 34% of the cases are diagnosed in advanced stages, presenting a significantly low life expectancy.

## Introduction

Endometrial cancer represents 6% of the cancer cases in women at the world level, in the developed countries representing the most frequent form of genital cancer. Besides the genital area, the frequency of appearance of endometrial cancer is situated below the breast, colon, and lung cancers. The maximum incidence is registered in post menopause, between 60 and 64 years old, but an increase in the affection of premenopausal women has also been registered. The incidence and mortality rate of endometrial cancer has been registering an increasing trend both in Romanian and in the whole world. 

## Aim

The analysis of the diagnosis approach of endometrial pathology in the University Emergency Hospital in Bucharest. 

## Material and Method

The current study is descriptive and combines techniques of quantitative research on main data with analyses of secondary data taken from national and international databases together with the specialty literature journals (critical evaluation). In order to achieve the objective, a descriptive quantitative analysis was undergone during 2011 and 2014. The cases analyzed were obtained from the registers and histopathological results emitted by the Service of Pathological Anatomy and also from the observation charts of the Departments of Obstetrics-Gynecology I, II and III, of the University Emergency Hospital Bucharest. The analysis included patients who addressed the University Emergency Hospital Bucharest during 2011 and 2014, and, who were analyzed based on the following criteria: age, environment, histologic sample used for the diagnosis of endometrial pathology, intraoperative staging of the endometrial cancer, and degree of tumor differentiation. For the statistical analysis realized in the current paper, intensive indexes (frequency) or extensive indexes (structure) were calculated, the study being structured as a retrospective analysis. The testing of the statistical significance was done by applying λ2 (chi-square) test, while respecting all the validation conditions of the test. 

## Results and Discussions

The cases presented in the Anatomy Department of the Emergency University Hospital in Bucharest were analyzed. They presented a positive anatomopathological diagnosis for endometrial cancer or previous/ associated lesion of cancer, respectively simple or complex endometrial hyperplasia, with or without atypias (HsfA, HcfA, HAS and HCA) or endometrial polyp. The classification of hyperplasias was done based on the WHO 1994 classification, updated in 2003 by the Society of Gynecologic Pathologists [**1**,**2**]. During the 4 years follow up, 826 patients with a positive anatomopathological diagnosis of endometrial cancer or previous/ associated lesion were registered in the AP Laboratory. The distribution according to calendar years is highlighted in **Table 1**. It can be noticed that the tendency of the annual number of cases is rising, according to the national, European and world trend. The raise of the incidence of endometrial pathology can be ascribed to the growth in the economical development, the growth in the rate of obesity and the incidence of metabolic syndrome, the growth in the care of the family physicians and the gynecologists regarding the investigation of the cases of abnormal uterine bleeding, as well as the growth of the information level of the patient, which has, as a result, the improvement of the addressability. 

**Table 1:** Distribution of the patients according to calendar years

Year	Total frequency	Relative frequency (%)
2011	163	19.7
2012	126	15.3
2013	300	36.3
2014*	237	28.7
Total	826	100.0
*Only the cases registered in the first 11 months were considered		

**Fig. 1:**
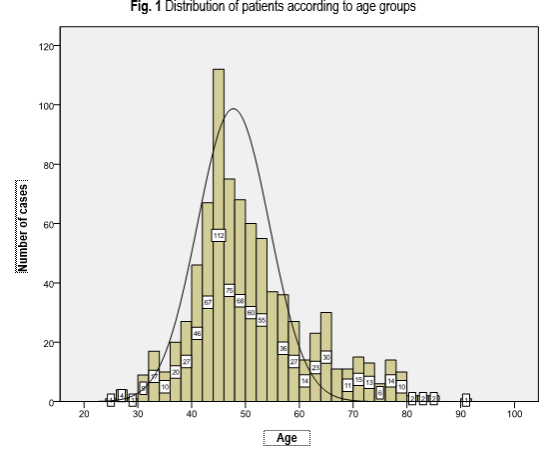
Distribution of patients according to age groups

Among the actuarial characteristics, age and residential area of the patients were analyzed. The medium age of the patients was 50.51 ± 10.924 years, and the median age was 48 years. The youngest patient was 25 years old and the oldest was 91 years old. The youngest patient suffering from endometrial cancer was 30 years old. The variation coefficient of the studied series was 21.62%, suggesting o moderate (acceptable) dispersion. The patients’ distribution according to age is presented in **Fig. 1**. 

From the point of view of the residential area, 72.3% of the patients (n=597) came from urban area. Given that 45% of the women live in the rural area and the access to the clinic is not subjected to the principle of territorialization, the analysis of the variable residential area, with the evidencing of the predominance of the urban area, raises the equity problem, as far as the access to services of the women in rural area is concerned, who, seem to be farther from the medical services from the geographical point of view and, possibly, from the economic and cultural one.

The type of the tumor examined from the anatomopathological point of view is shown in **Table 2**. The hysterectomy piece with bilateral adnexectomy in the resection piece, the resected or biopsied lymphatic ganglions in the surgical staging and the biopsy fragments of the suspected areas or the cytoreduction fragments of the secondary determinations intraoperatory observed, were included. The endometrial fragments were obtained by dilation and biopsy curettage sometimes also performed with a hemostatic purpose. In addition, the statistical data indicate the right dilation and curettage as the main method used to perform the endometrial biopsy, on which the certainty etiologic histopathological diagnosis was made in the case of 68.4% of the patients with endometrial pathology. 

**Table 2:** Type of tumor for examination

Type of tumor	Absolute frequency	Relative frequency (%)
Resection tumor	223	27.0
Endometrial fragments	566	68.4
Aspirated fragments	3	0.4
Endometrial polyps (other)	34	4.1
Total	826	100

The aspirated tumors represent endometrial fragments obtained as a result of biopsy by aspiration; the 3 cases represent 0.4% of the material which was histopathologically analyzed due to the lack of availability of Pipelle aspiration disposable devices and the lack of experience and trust in using them by the gynecologists. The extremely limited use of aspiration endometrial biopsy determines the inoperability of the clinic regarding the exploration of the endometrial pathology, invasiveness, grown rate of complications and the costs for hospitalization, being important factors associated to the first intention practice of dilation and biopsy uterine curettage, currently considered the “gold standard” of uterine cavity exploration. In the 5 procedures performed during the studied period, 2 had insufficient material for the histopathological study of the endometrium, and the other 3 were correctly diagnosed: 1 case of endometrial polyp, although the focal character of the lesion decreased the general sensitivity of the screening technique, 1 case of simple hyperplasia without atypias and 1 case of complex hyperplasia without atypias. The statistics includes the “polyps” subcategory, which were extirpated by torsion and histopathologically confirmed as endometrial polyps (fibro glandular, atrophic, hyperplasic with cystic atrophy). Regarding the tumors, a special case was introduced, represented by the relapse of an endometrial carcinoma on a vaginal stamp. The analysis excluded all the endometrial biopsies in which the histopathological examination revealed physiologic modifications of the endometrium during the period (endometrium under proliferative stimulus, endometrium under estrogenic impulse, endometrium under medio-proliferative stimulus), as well as the cases of dysfunctional endometrium and the ones which presented different forms of metaplasia (mature/ immature squamous metaplasia, hobnail metaplasia of luminal epithelium). The endometrial biopsies resulted from the biopsy curettage of the uterine cavity without any histopathological element of endometrial pathology, and excluded from the analyzed statistics, represented the evaluation procedures of the endometrium, performed during the mandatory protocol of preoperatory evaluation of the patients with uterine leiomyomas, in the Obstetrics-Gynecology Clinics in the Emergency University Hospital in Bucharest.

Patients who were subjected to endometrial biopsy, mostly had abnormal uterine bleedings as a main reason for hospitalization; a low number of patients were represented by the anomalies present in the cervical cytology examination and also a low number of patients were subjected to exploration by biopsy, consecutive to the accidental CT discovery of an endometrium with a thickness of > 4 mm post menopause, or thickened, nonhomogeneous, with a suggestive echographic aspect of hyperplasia or premenopause endometrial polyp, in asymptomatic patients [**3**-**5**]. None of the procedures had as a main purpose the endometrial cancer screening in a patient with a diagnosis or family history of Lynch syndrome, only one case of endometrial biopsy for hyperplasia being recorded, and having a histopathological diagnosis of persistent disease, respectively simple hyperplasia without atypias. 

From the point of view of the anatomopathological diagnosis, endometrial cancers and precursor/ associated lesions of hyperplasia type (with or without atypias) and polyps were taken into consideration. Their frequency in the studied population is presented in **Table 3**. 

**Table 3:** Frequency of the anatomopathological diagnoses in the studied group

Disease	Absolute frequency	Relative frequency
Hyperplasia without atypias	386	46.7
• Simple hyperplasia without atypias	282	34.1
• Complex hyperplasia without atypias	104	12.6
Hyperplasia with atypias	68	8.2
• Simple hyperplasia with atypias	14	1.7
• Complex hyperplasia with atypias	54	6.5
Polyps	183	22.2
Endometrial carcinoma	157	19,0
Associations	32	3.9
Total	826	100.0

Hyperplasias represented half of the pathology (54.9%, n=454), most of them being without atypias. The endometrial carcinoma was identified in 19% of the patients, and 3,9% of the cases presented associations of the endometrial pathological entities studied. Taking into account the global incidence of endometrial hyperplasia (133/ 100000 women) and of endometrial cancer (8,2/ 100000 women), the ratio of these pathologies is of approximately 16/ 1, and, regarding the studied group, the cases of hyperplasia and endometrial cancer were of 3/ 1. The difference is explained by the Multidisciplinary Emergency Hospital addressing characteristics, to which serious cases that have a histopathological confirmation often done in advance, which need complex surgeries, and the addressing to young women with chronic abnormal uterine bleeding mainly targets the ambulatory system, are mainly directed to other medical units.

The literature data, according to which the incidence of endometrial polyps in biopsies or hysterectomy tumors is situated between 10 and 24%, are confirmed by our study, in which polyps represent 22,2% of the studied endometrial pathology.

**Fig. 2:**
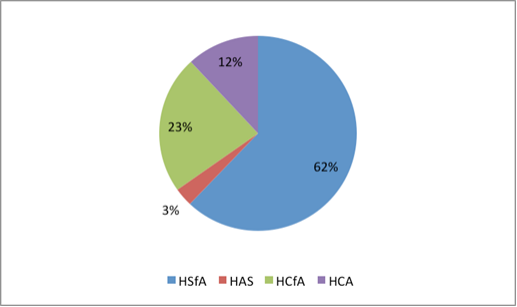
Structure of hyperplasia cases according to its type

Among the cases of hyperplasia without atypias, most were simple (62%), the rest being complex, while in atypical hyperplasias, most were complex (12%), an aspect in accordance with the date in literature. The structure of hyperplasia cases is presented in **Fig. 2**.

The cases with associated affections are detailed in **Table 4**. There were 31 cases of endometrial polyps associated with hyperplasia in the analyzed group, among which 12 cases of polyp + hyperplasia with atypias, 19 cases of polyp + hyperplasia without atypias. Beside the risk factors common for polyps and hyperplasia, their association highlights the risk potential endometrial polyps present for the development of endometrial cancer, especially post menopause. 

**Table 4:** Frequency of associated affections on categories

Diseases combinations	No. of cases
Complex hyperplasia without atypias + polyp	12
Simple hyperplasia without atypias + polyp	7
Simple atypical hyperplasia + polyp	7
Complex atypical hyperplasia + polyp	5
Simple hyperplasia without atypias + complex atypical hyperplasia	1
Endometrial carcinoma + complex atypical hyperplasia	1
Endometrial carcinoma + complex hyperplasia without atypias	1
Endometrial carcinoma + polyp	1
Total	35

Moreover, in the studied group of patients, the association of hyperplasia without atypias with atypical hyperplasia or endometrial carcinoma is present, supporting the etiopathogenic affiliation of the lesion, being demonstrated that 37% of the cases with atypia hyperplasias coexist with endometrial cancer. 1 case of endometrial polyp associated with carcinoma, was evidenced in the group of patients at this level, without a histopathological specification of the existence of any malignity at the level of the polyp or in the surrounding endometrial tissue, the specialty literature arguing that the risk of endometrial cancer is 9 times higher in women with endometrial polyps compared to the general population [**6**].

Analyzing the resection tumors, the observation of the association of endometrial pathology with leiomyomas is very important from the clinical point of view, and, beside the partially common etiopathogenic affiliation, these 16 cases of uterine fibroids (among which 11 were associated to simple endometrial hyperplasia, one to atypical hyperplasia and 4 to polyps), plus a case of leiomyosarcoma, are the ones to which endometrial pathology was randomly found histopathologically intraoperatory or postoperatory, representing false negative results of the procedures of endometrial biopsy, performed postoperatory. 

Starting with the evidence provided by the specialty literature, according to which premenopause women with endometrial cancer present a high risk of primary synchronous disease or ovarian metastasis, appreciated at 25%, the association of the endometrial pathology with the ovarian pathology, although without a statistical significance, needs to be recorded in the studied histologic bulletins, the following aspects being present: 

• Simple hyperplasia without atypias + ovarian mucinous cystadenoma (49 years old); 

• Simple hyperplasia without atypias + ovarian serous carcinoma (43 years old); 

• Simple hyperplasia without atypias + ovarian papillary adenocarcinoma (78 years old); 

• Simple hyperplasia without atypias + ovarian serous cystadenoma (63 years old); 

• Simple hyperplasia without atypias + ovarian serous cystadenoma (43 years old); 

• Simple hyperplasia without atypias + ovarian serous cystadenoma (53 years old); 

• Simple hyperplasia without atypias + ovarian serous cystadenoma + leiomyoma (48 years old);

• Complex hyperplasia with atypias + ovarian serous adenocarcinoma (55 years old); 

• Polyp + ovarian papillary cystadenoma (59 years old); 

• Polyp + ovarian cyst fibroma (53 years old);

• Endometrial carcinoma stage II + ovarian carcinoma stage III B (64 years old). 

The association of ovarian pathology, especially malignant, with the endometrial one, mostly in young patients, can suggest genetic Lynch syndrome. It is advisable that this category of patients benefit from a genetic consultation and specialty clinical investigations.

**Fig. 3:**
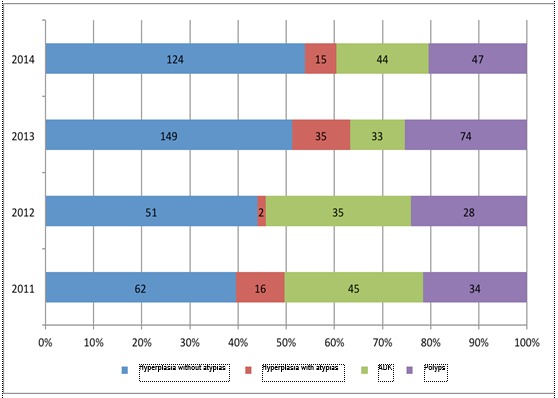
Proportion of the disease cases according to calendar years

After that, the time and person characteristics were analyzed for each group of diseases, mentioning that the three cases of endometrial carcinoma associated with hyperplasia or polyps have been included in the group of patients with endometrial carcinoma and the rest of the diseases associations were not taken into consideration (N=794). The proportion of each pathology according to the calendar years is evidenced in **Table 5** and also in **Fig. 3**. 

**Table 5:** The characteristics of time and person for the entities of the studied endometrial pathology

	2011		2012		2013		2014	
	No.	%	No.	%	No.	%	No.	%
Hyperplasia without atypias	62	39%	51	44%	149	51%	124	54%
Hyperplasia with atypias	16	10%	2	2%	35	12%	15	7%
Endometrial carcinoma	45	29%	35	30%	33	11%	44	19%
Polyps	34	22%	28	24%	74	25%	47	20%
Total	157	100%	116	100%	291	100%	230	100%

The proportion of simple hyperplasias without atypias was growing in the time interval analyzed, doubling in 2014 as compared to 2011. The share of endometrial polyps also registered a growth in the endometrial pathology during the period 2011-2014, which was explained by the growth of the echographic diagnosis of this entity together with the extension of Doppler color evaluation by more and more gynecologists in the University Emergency Hospital, in Bucharest. The difference in the appearance of each pathology was statistically significant (p<0,001) (p = 0.000003771, Chi2 test).

Moreover, the lowest proportion of the cases of endometrial carcinoma was evidenced in 2013 (11%), and the highest in 2012 (30%), the annual percentage differences being statistically significant (p<0,001) (0.000002427, Chi2 test). The rate fluctuations can be partially attributed to the modifications of the teams gynecologist – oncologist surgeon, who usually manage these cases in the University Emergency Hospital in Bucharest, as well as the retirement of some physicians who are specialized in oncologic gynecology. 

The analysis of age for each pathology evidences particularities of the studied group compared to the data in literature (**Table 6**). The most important study developed on a period of 18 years, regarding the incidence of endometrial hyperplasias, published in 2009 by Reed et al. [**7**], evidenced a medium diagnosis age of hyperplasias between 50 and 54 years old, the hyperplasias without atypias of the study group having a medium diagnosis age lowered to 47 years old, while atypical hyperplasias appeared according to the study with a maximum rate between 60 and 64 years old, the analyzed group presenting a medium age of this pathology which was also low, respectively 53 years old. Among the 68 cases of atypical hyperplasia, 19 cases were diagnosed between 40 and 50 years old (**Fig. 5**). Polyps represent the pathology with the most precocious age of occurrence, 25 years old. 

**Table 6:** Age analysis for each pathology

		Hyperplasia without atypias	Hyperplasia with atypias	ADK	Polyps
Medium		47.07	53.07	64.06	45.60
95% Confidence interval for medium	Inferior limit	46.40	51.10	62.47	44.07
	Superior limit	47.74	55.04	65.65	47.13
Median		46.00	53.00	64.00	44.00
Variant		44.538	66.218	101.824	110.065
Std deviation		6.674	8.137	10.091	10.491
Minimum		27	32	30	25
Maximum		79	77	91	79
Amplitude		52	45	61	54

**Fig. 4:**
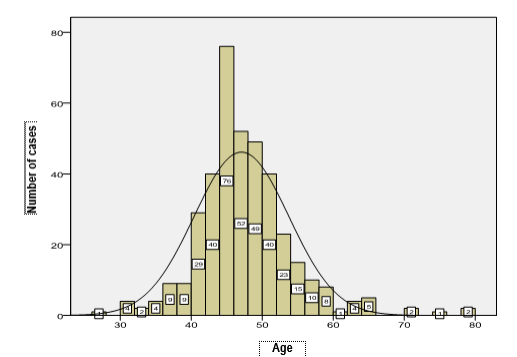
Distribution of cases of hyperplasias without atypias according to age

**Fig. 5:**
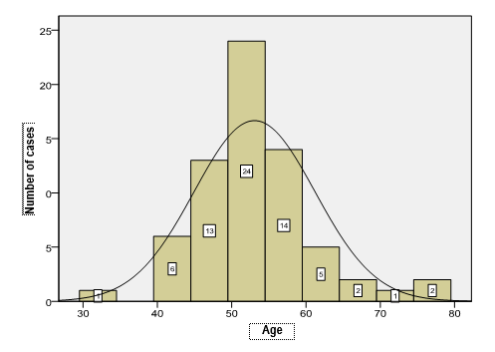
Distribution of cases of hyperplasia with atypias according to age

**Fig. 6:**
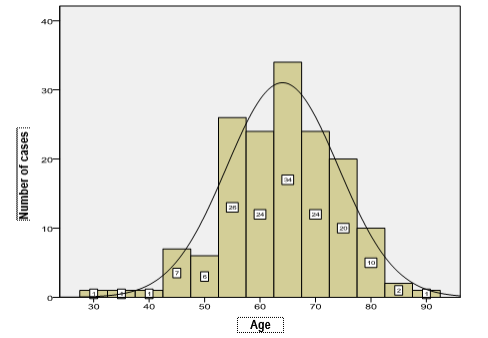
Distribution of ADK cases according to age

**Fig. 7:**
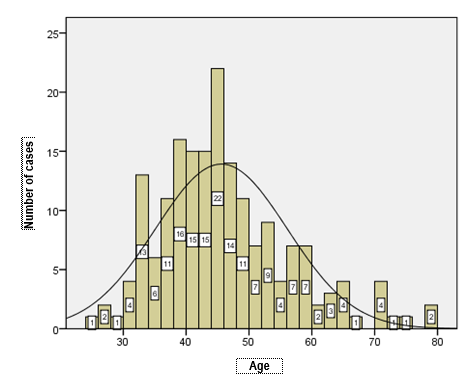
Distribution of cases of polyps according to age

Further, the distribution of age in each pathology was presented (**Fig. 4**-**7**). The medium age of occurrence of endometrial cancer is 64 years old, but the 3 cases between 30 and 40 years and the 11 cases diagnosed in women below 50 years old were the most concerning. The affiliation of these tumors to Lynch syndrome was not tested from an immunohistochemical point of view due to a lack of a standard protocol. Most of the cases of endometrial cancer occur between 55 and 75 years old, because after 52 years old, the raise of incidence is abrupt. 

Endometrial polyps also occur in unusually young women, registering the maximum incidence around 45 years old, a high amount of cases being registered in post menopause, when the risk of polypoid growth and malignization is very high. Although the specialty literature affirms the rarity of the development of polyps before 40 years old, the scientific data and the current practice demonstrate their high frequency of occurrence between 30 and 45 years old.

The analysis of the age variable for each disease revealed a Gaussian distribution for endometrial carcinoma (Kolmogorov-Smirnov test, p=0.200), for all the other pathologies the distribution of age being significantly different compared to the normal one (Kolmogorov-Smirnov test, p<0.05).

**Fig. 8:**
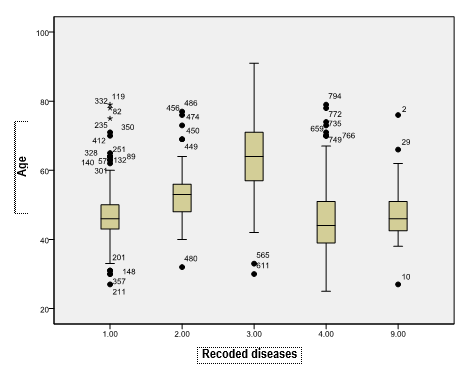
Median age according to the pathology groups (including extreme values)

**Fig. 9:**
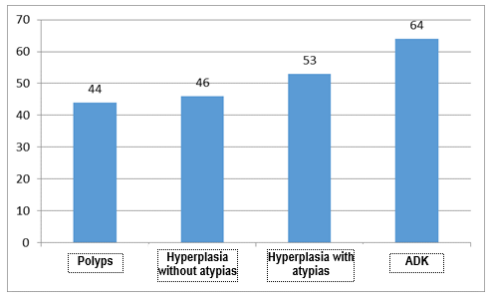
Median age according to each pathology

The median age according to the pathology was of 44 years old for polyps, 46 years old for hyperplasia without atypias, 53 years old for hyperplasia with atypias and 64 years old for adenocarcinoma, the raise of the median being significant from one pathology to the other (Mann Whitney U test, p<0.05 – the comparison of the medians was chosen due to the non-Gaussian distribution of the age variable). The median age of each group of diseases is presented in **Fig. 8**,**9**. 

The median ages are significantly different in groups of two (Mann Whitney U test):

• Hyperplasia without atypias compared to polyps (p<0.001);

• Hyperplasia without atypias compared to atypical hyperplasia (p<0.001);

• Atypical hyperplasia compared to endometrial carcinoma (p<0.001).

**Fig. 10:**
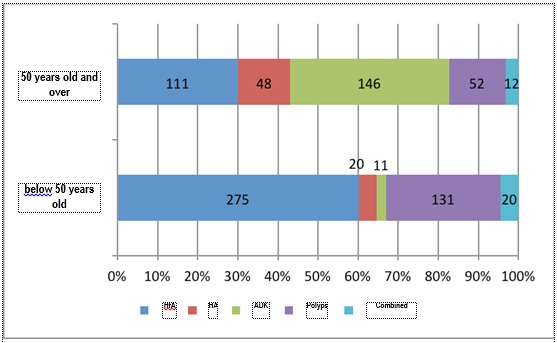
The distribution of anatomopathological diagnoses according to age groups

**Fig. 11:**
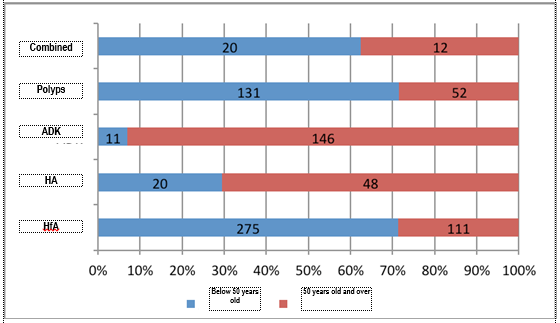
Analysis of the pathology specific to age

Taking menopause as a mark, whose medium age of installation is 51 years old, and the age considered by the international specialty societies as a threshold of high risk for the occurrence of genetic forms of cancer, the endometrial pathology was divided into two periods: below 50 years old, including the women at fertile age and over 50 years old, women in peri and post menopause (**Table 7**). Analyzing the distribution of the anatomopathological diagnoses according to these two age groups (**Fig. 10**,**11**), it was noticed, just as expected, that benign pathology is predominant in premenopause and the malignant one in post menopause. What is interesting is the fact that although endometrial cancer represents, according to the specialty literature, only 10% of the bleeding causes in post menopause, in the analyzed group, the rate of this pathology was much higher (39.6%), out of the total number of histopathological results in the study. 

**Table 7:** The analysis of the patients according to age groups (<50 years old and ≥ 50 years old)

Age group	Absolute frequency	Relative frequency (%)
Below 50 years old	457	55.3
50 years old and over	369	44.7
Total	826	100.0

The same data analyzed from another perspective evidenced the fact that the polyps and te simple hyperplasia without atypias are attributed to the fertile age, while the endometrial cancer is specific for the perimenopause and post menopause period. The decrease tendency of the age of occurrence of endometrial cancer is reflected in the 7% cases diagnosed below 50 years old, but mostly in the 29,4% cases of atypical hyperplasia below 50 years old, a histopathological entity whose risk of progression to cancer is of 29% and whose risk of coexistence with it reaches 37% [**8**]. 

The addressability for each type of disease according to the residence areas was compared in **Table 8**. The rate of patients from the urban area was significantly higher. 

**Table 8:** Proportion of cases according to the residence areas for each disease

	HfA	HA	ADK	Polyps	Total
Urban	76%	75%	69%	68%	72%
Rural	24%	25%	31%	32%	28%

The analysis of the type of medical treatment for each diagnosis is presented in **Table 9**. It was noticed that the endometrial biopsy was the predominant method of diagnosis for all the precursor or associated lesions, while the tumor was mostly the same for the diagnosis of endometrial cancer. Except for the numerically reduced cases, in which cancer was histopathologically determined unexpectedly on the hysterectomy tumor, the rest of the cases prove the performing of endometrial biopsy in other sanitary units. The lack of continuity and the dispersion of the cases between different diagnosis stages performed in different sanitary units are only detrimental to the patient and the medical system, implying high costs and the impossibility of centralizing and following the cases, the necessity of creating common databases in all the sanitary units and specific to the patient being a must. 

**Table 9:** Analysis of the type of tumor for each diagnosis

	HfA		HA		ADK		Polyp		Combinations		Total	
	No.	%	No.	%	No.	%	No.	%	No.	%	No.	%
Tumor	76	19.7%	14	20.6%	110	70.1%	20	10.9%	4	12.5%	223	27.0%
Endometer	308	79.8%	54	79.4%	47	29.9%	129	70.5%	27	84.4%	565	68.4%
Aspirated	2	0.5%	0	0.0%	0	0.0%	1	0.5%	0	0.0%	3	0.4%
Other	0	0.0%	0	0.0%	0	0.0%	33	18.0%	1	3.1%	35	4.2%
Total	386	100.0%	68	100.0%	157	100.0%	183	100.0%	32	100.0%	826	100.0%
* The cases presenting combined affections and the ones for which the type of the tumor was not mentioned, were excluded from the analysis.												

**Fig. 12:**
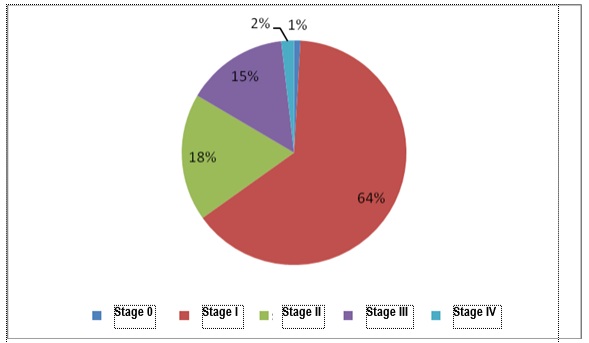
Structure of the cases of endometrial carcinoma according to stages

Endometrial cancer was evidenced in 157 patients. The diagnosis was established based on the tumor in 109 cases, these being studied further from the point of view of staging, in 48 cases the diagnosis being established based on endometrial fragments obtained through dilation and biopsy curettage. To the 69,4% of the cases which were histopathologically diagnosed based on the resection tumor, the surgical staging was available with the exception of the evaluation of the para-aortic ganglions which was not performed in 11,93% of the cases. The staged cases are presented in **Table 10** and the structure of the cases of endometrial carcinoma on stages and residence areas is presented in **Fig. 12**,**13**. Staging was performed according to FIGO/ TNM 2001 classification [**9**], which in contrast with the current classification includes the following: 

T Category 

Tis Tumor in situ

T1a Tumor limited to the endometrium 

T1b Tumor invades less than half of the myometrium 

T1c Tumor invades half or more of the myometrium 

T2a Endocervical glandular invasion 

T2b Cervical stromal invasion 

N Category

N0 Regional lymphatic ganglions without metastases 

N1 Regional lymphatic ganglions with metastases present in the pelvic ganglions and/ or the para-aortic ganglions 

**Table 10:** Analysis of the type of tumor for each diagnosis

Stage	T	N	M	Absolute frequency			Relative frequency (%)		
				urban	rural	total	urban	rural	total
Stage 0	Tis	N0	M0	1	0	1	1%	0%	1%
Stage 1A	T1a	N0	M0	5	1	6	7%	3%	6%
Stage 1B	T1b	N0	M0	26	14	40	36%	39%	37%
Stage 1C	T1c	N0	M0	17	7	24	23%	19%	22%
Stage IIA	T2a	N0	M0	9	4	13	12%	11%	12%
Stage IIB	T1b	N0	M0	3	4	7	4%	11%	6%
Stage IIIA	T3a	N0	M0	3	0	3	4%	0%	3%
Stage IIIB	T3b	N0	M0	1	0	1	1%	0%	1%
Stage IIIC	T1,T2,T3	N1	M0	6	6	12	8%	17%	11%
Stage IVA	T4	Any N	M0	1	0	1	1%	0%	1%
Stage IVB	Any T	Any N	M1	1	0	1	1%	0%	1%
Total				73	36	109	100%	100%	100%

The most important element highlighted by the analysis of the stages of the cases is that at present, in a group of 109 cases of endometrial cases, the diagnosis of the disease in stage IA (tumor limited to the endometrium) represents 5,5% of all the cases of endometrial cancer and, it is situated below the reported global level. If we refer to the diagnosis of the disease in its limited stage to the uterus (stage IA, IB and IC), the rate is of 64,2%, situated below the rate of 68% reported by U.S.A. for a disease diagnosed in stage I, and whose survival rate at 5 years is of 96% [**10**]. 34,9% of the patients are diagnosed in advanced stages of the disease, the life expectancy being highly lowered. 

Although the analysis according to residence areas evidenced that for the rural area there is a higher proportion of diagnosed advanced cancers (38.9%, compared to 32.9% in urban area), the difference was not statistically significant (p=0.535, Chi2 test). Moreover, the addressability to the urban area in an early stage of the disease is obviously higher. 

**Fig. 13:**
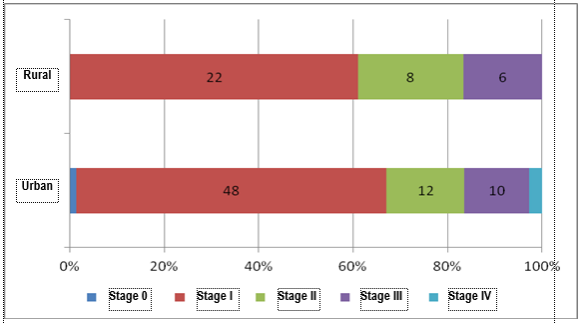
Structure of cases of endometrial carcinoma according to stages and residence areas

**Table 11:** Histological classification (WHO) of cases of endometrial carcinoma

Histological classification of endometrial cancer	Absolute frequency		
	urban	rural	total
Endometrioid adenocarcinoma	55	32	87
Variants	4	1	6
• With squamous differentiation	16	5	21
• Villoglandular	6	6	12
• Secretory	0	0	0
• With ciliated cells	0	0	0
Mucinous adenocarcinoma	2	0	2
Serous carcinoma	1	3	4
Clear cells carcinoma	2	2	4
Mixed carcinoma	1	1	2
Squamous carcinoma	3	1	4
Transitional cell carcinoma	0	0	0
Small-cell carcinoma	0	0	0
Undifferentiated carcinoma	2	1	3
Others	9	4	13

From the histopathological point of view, this was mentioned in 100% of the cases (**Table 11**). The endometrioid adenocarcinoma represents the most encountered histopathological form. From the histopathological types that place the patients in the high-risk category for recurrence and persistence of the disease, we could notice the presence of 4 cases of squamous carcinoma and 4 cases of clear cells carcinoma. The mixed carcinoma was present in 2 cases, this pathological type combining at least 10% of both types I (endometrioid/ rarely mucinous) and type II (mostly serous/ rarely clear cells). The category of rare tumors was represented in the studied group by 3 cases of undifferentiated carcinoma and a surprisingly high number (N=4) of cases of squamous carcinoma. Because they were not included in FIGO staging, we considered it important to separately mention the 2 cases of sarcoma: case 1: endocavity sarcoma, patient from the rural area, 63 years old, case 2: leiomyosarcoma moderately differentiated with limpho-ganglionar and epiploic metastases, patient from the rural area, 53 years old. 

Regarding the degree of tumor differentiation (**Table 12**), it was established for 68,15% of the cases (n=107), the degrees 1 and 2 being the most encountered (88%, n=94), according to the literature data. Conjoining the presence of 17 cases of serous, undifferentiated, mixed, squamous, clear/small/transition cells carcinoma, among which 13 being of 3rd degree of differentiation, it results that 10,82% of the patients in the high risk category present aggressive histopathological characteristics, need adjuvant chemotherapy and have a bad prognosis, the mortality risk due to the disease being of 30-40% in 5-10 years. 

**Table 12:** Degree of differentiation of adenocarcinoma

Degree of differentiation		Absolute frequency			Relative frequency (%)		
		urban	rural	total	urban	rural	total
G1	≤ 5% pattern of solid non-squamous/ non morular growth (highly differentiated)	42	18	60	59	50	56
G2	6-50% pattern of solid non-squamous/ non morular growth (moderately differentiated)	21	13	34	30	36	32
G3	> 50% pattern of solid non-squamous/ non morular growth (weakly differentiated)	8	5	13	11	14	12
TOTAL		71	36	107	100	100	100

**Fig. 14:**
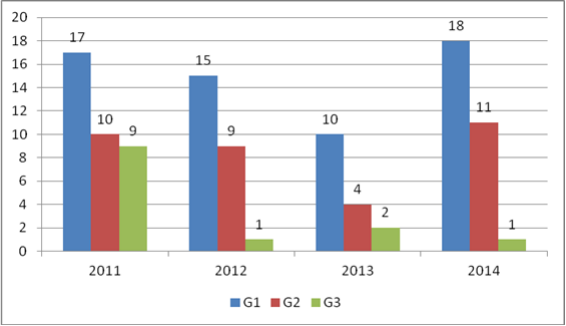
Situation of adenocarcinomas according to the degree of differentiation on calendar years

The distribution on calendar years of the tumor degrees of differentiation highlights an encouraging growing tendency in time of the high and medium differentiated forms, as opposed to the 3rd degree (**Fig. 14**). 

**Fig. 15:**
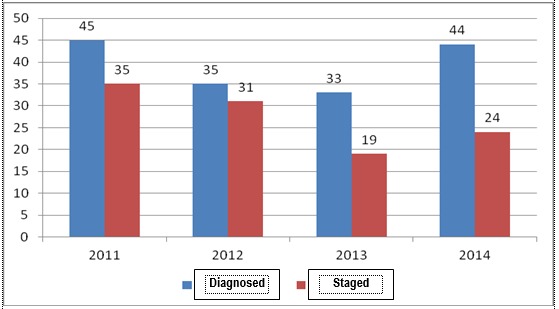
Situation of endometrial carcinomas no matter the diagnosis and staging, according to calendar years

**Fig. 16:**
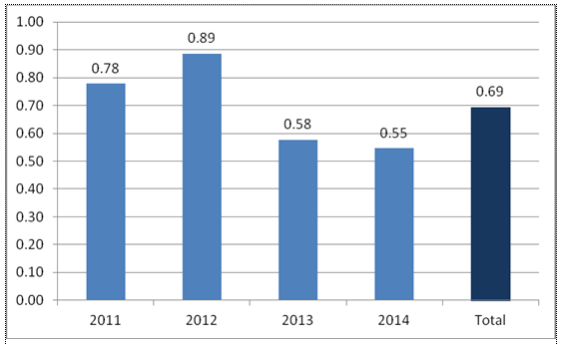
The report staged adenocarcinomas vs. diagnosed, according to calendar years

In the analyzed medical center it could be observed that, generally, in every year of the last 4 (**Fig. 15**,**16**), between 55% and 89% of the cases have been diagnosed according to the tumor and stage, with a lowering tendency of the treated cases, the analysis highlighting an aspect, whose causes must be established and improved as soon as possible, the rest of the percentage representing the cases diagnosed in the University Emergency Hospital in Bucharest and treated or programmed for surgery in other medical centers. 

## Conclusions

At the national level, the cases of endometrial cancer generally and stage I of the disease in particular, most frequently come from the urban area compared to the rural area and most frequently from persons with a higher level of education, which raises the problem of the scratchy cultural level and social inequity regarding the access to the health services. The risk factors for endometrial cancer are generally known; having as a common denominator the endogenous or exogenous hyperestrogenism, inadequately countered as far as progesterone is concerned and also genetic syndromes which are transmitted hereditarily. The lesion precursor for most of the cases of endometrial cancer is endometrial hyperplasia. The most important syndrome which should determine the visit the physician is the abnormal uterine bleeding. Based on this syndrome, 66% of the cases of endometrial cancer in the stage limited to the uterus are diagnosed in Romania. However, 34% of the cases are diagnosed in advanced stages, which present a significantly reduced life expectancy. The most encountered histopathological type is the endometrioid adenocarcinoma with a high or moderate degree of differentiation. The natural evolution of the disease is generally slow and the relatively favorable prognosis depends on the stage of the disease at the moment the patient presents to the physician, the histologic type and the tumor degree. The main method of diagnosis of endometrial cancer in Romania is the endometrial biopsy by dilation and uterine curettage. Compared to the biopsy by aspiration, which represents the first line of exploration in U.S.A. and in most of the European countries, due to its reliability, high degree of acceptance, reduced costs and availability to be used in an ambulatory system, having a high accuracy verified by multicentric controlled studies and the accomplished universal experience, uterine biopsy curettage, presupposes a minimum hospitalization period of minimally a day, extra costs, anesthesia, significantly high risks, discomfort, a decrease in the addressability, which reduces even more in cases in which consultations must be repeated, staged, due to the long period of time necessary to obtain the histopathological result of the biopsy. Transvaginal ultrasound represents the main method of selecting the abnormal uterine bleedings in post menopause, which must be subjected to endometrial biopsy, reducing on reliable criteria the amount of the useless invasive maneuvers to 40%. It also directs the optimum further investigation and excludes the other structural causes of abnormal uterine bleeding and annexes pathology. The standardized criteria of endometrial exploration available from 2010 must be integrated in the medical practice in Romania. In our opinion, and in concordance with the current world tendency, in the context of the large availability of the high resolution devices and the extension of the technical abilities of ultrasound use by the the modern generations of gynecologists, the use of transvaginal ultrasound as a first line of exploration of the endometrium, especially when its pathology clinically manifests, represents an optimum cost-benefit approach, it is minimally invasive and extremely useful in directing and prioritizing the differential diagnosis of the cases and also in evaluating the extension of the disease. FIGO staging was revised starting with 2010, but its present formula must be included in the guide of the Ministry of Health with the theme “Endometrial Cancer” and also generally integrated in the current national medical practice. Endometrial cancer screening is not recommended to the general population. Until present, there has not been any scientific evidence to justify the appliance of screening in the high-risk population, except for Lynch syndrome. The screening method is endometrial biopsy. The annual high amount of patients who present to the University Emergency Hospital in Bucharest with an abnormal uterine bleeding has allowed the accomplishment of a vast experience in studying the causes of this pathology, confirmed in most of the cases by the histopathological examination. 
